# Molecular mechanisms of HIV-1 persistence in the monocyte-macrophage lineage

**DOI:** 10.1186/1742-4690-7-32

**Published:** 2010-04-09

**Authors:** Valentin Le Douce, Georges Herbein, Olivier Rohr, Christian Schwartz

**Affiliations:** 1 INSERM unit 575, Pathophysiology of Central Nervous System, Institute of Virology, rue Koeberlé, 67000 Strasbourg, France; 2 IUT Louis Pasteur de Schiltigheim, University of Strasbourg, 1 Allée d'Athènes, 67300 Schiltigheim, France; 3 UPRES EA 3186, Pathogens and Inflammation, CHU of Besançon, University of Franche-Comté, Department of Virology, 25030 Besançon, France

## Abstract

The introduction of the highly active antiretroviral therapy (HAART) has greatly improved survival. However, these treatments fail to definitively cure the patients and unveil the presence of quiescent HIV-1 reservoirs like cells from monocyte-macrophage lineage. A purge, or at least a significant reduction of these long lived HIV-1 reservoirs will be needed to raise the hope of the viral eradication. This review focuses on the molecular mechanisms responsible for viral persistence in cells of the monocyte-macrophage lineage. Controversy on latency and/or cryptic chronic replication will be specifically evoked. In addition, since HIV-1 infected monocyte-macrophage cells appear to be more resistant to apoptosis, this obstacle to the viral eradication will be discussed. Understanding the intimate mechanisms of HIV-1 persistence is a prerequisite to devise new and original therapies aiming to achieve viral eradication.

## Introduction

Human immunodeficiency 1 (HIV-1), identified in 1983 [1], remains a global health threat responsible for a world-wide pandemic. Several advances have been made in curing acquired immune deficiency syndrome (AIDS) since the introduction of the highly active antiretroviral therapy (HAART) in 1996. AIDS pandemic has stabilized on a global scale in 2008 with an estimated 33 million people infected worldwide (data from UN, 2008). Even if an effective AIDS vaccine is still lacking, the introduction of HAART greatly extended survival. This therapy can reduce plasma virus levels below detection limits (≤ 50 copies/ml). It induces a biphasic decline of HIV-1 RNA with a rapid decline of infected CD4+ T cells (half life 0.5 day) followed by a decline originating from infected tissue macrophages (half life 2 weeks) [2]. However, with very sensitive methods [3,4], a residual viremia is still detected in patients on HAART. Moreover, HIV RNA returns to a measurable plasma level in less than two weeks when HAART is interrupted [5,6]. These observations suggest that even long term suppression of HIV-1 replication by HAART cannot totally eliminate HIV-1, the virus persists in cellular reservoirs because of viral latency, cryptic ongoing replication or poor drug penetration [7-9]. In fact, the persistence of infection is not so surprising since, from an evolutionary point of view, this is the best form of adaptation of viruses to the host environment. There are essentially two theories of persistent infection: latency and ongoing replication. Latency is best described as a lack of proviral gene expression. On the other hand, ongoing replication requires continuous viral expression without cytopathic effects. It is important to distinguish between the two possibilities since they call for very different therapeutic interventions. The theory of ongoing replication suggests that drug resistance to treatment may develop. In this case treatment intensification and the design of new anti HIV-1 molecules are needed in the long term. On the other hand, if viruses are released by burst from stable reservoirs, multi drug resistance does not develop, however HAART alone is ineffective. In this case new strategies are needed to purge the reservoirs, which in combination with HAART should be able to eradicate the virus in infected patients.

Resting memory CD4+ T cells are the major cellular and the best characterized reservoir in the natural host [7,10-13]. The presence of latent proviral HIV-1 DNA in this cell population has been undoubtedly proven [10]. But there are other reservoirs. Genetic studies showed that during rebound viremia (when HAART was interrupted) the virus could be detected from another reservoir than the CD4+ T cells [14-16]. It has been proposed that peripheral blood monocytes, dendritic cells and macrophages in the lymph nodes and haematopoitic stem cells in the bone marrow can be infected latently and therefore contribute to the viral persistence [17-22]. It is still debated whether or not viral persistence in these latter reservoirs is due to true latency or to low level ongoing replication [23,24].

In this review, we focus on the molecular mechanisms responsible for viral persistence in cells of the monocyte-macrophage lineage since they are believed to be an important source of HIV-1 [14,19]. Several features make cells from this lineage a potential HIV-1 reservoir. Contrary to CD4+ T cells, HIV-1 infection is generally not lytic for these cells [25,26]. The particles produced in macrophages are budding into intracytoplasmic compartments which may represent favored sites for HIV-1 assembly. [27,28] (see also the accompanying review from Benaroch et al). Mechanisms underlying HIV-1 budding that involved Gag and the ESCRT pathway, were recently reviewed [29]. Cells from monocyte-macrophage are also more resistant to cytopathic effects and they are able to harbor viruses for a longer period. It may arrive that infected tissue macrophages, such as microglial cells in the brain, produce viruses during their total lifespan [30]. Finally, a major obstacle for the eradication of the virus is that HIV-1 makes infected monocyte-macrophage cells more resistant to apoptosis. Understanding the intimate mechanisms underlying HIV-1 persistence in the monocyte-macrophage lineage will be needed to devise new and original therapies to achieve viral eradication.

## Evidence for the constitution of an HIV-1 reservoir by cells from the monocyte-macrophage lineage

**Figure 1:**
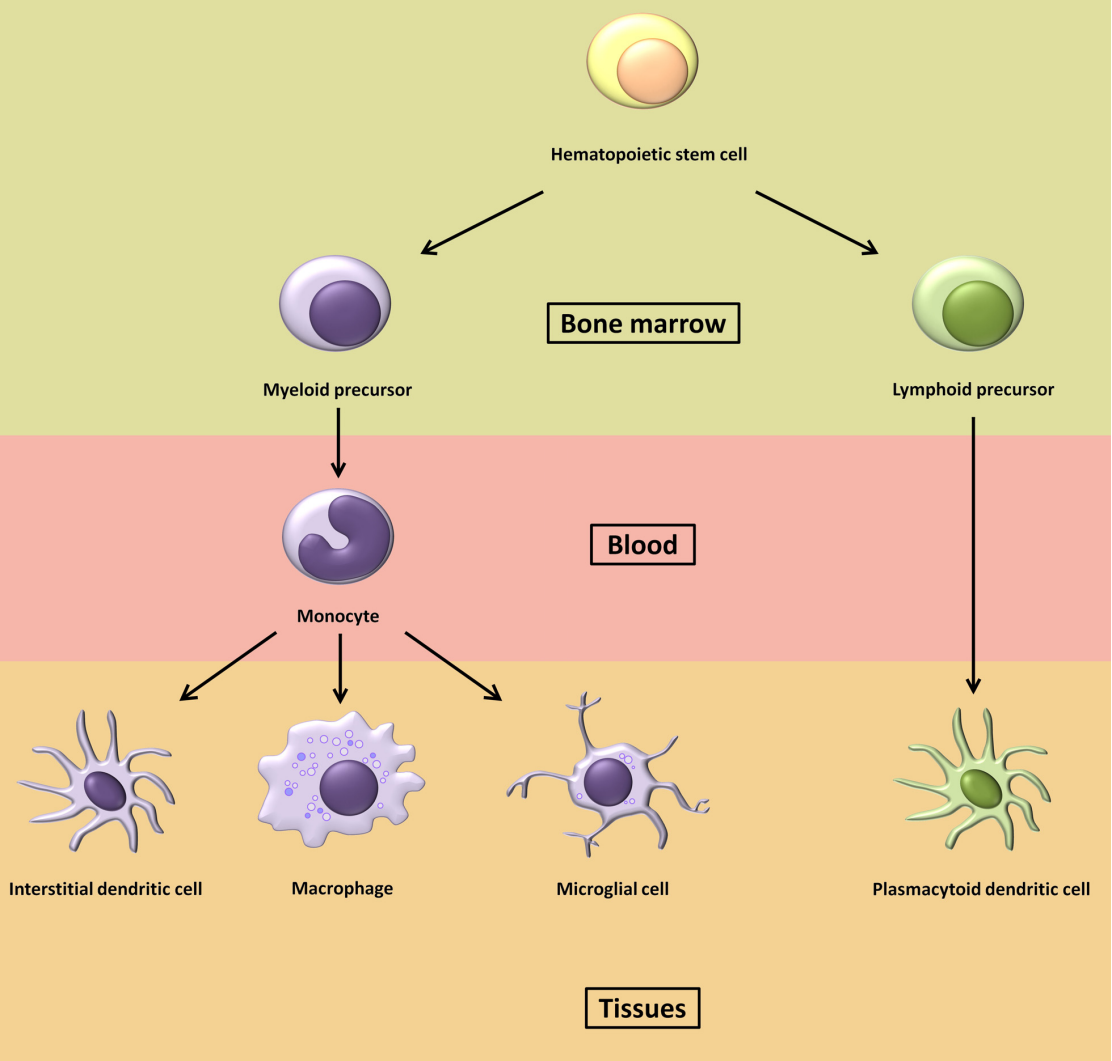
**monocyte-macrophage lineage**. All cells from the monocyte-macrophage lineage appear to derive from a same progenitor multipotent cell, the hematopoietic stem cell (HSC). The HSC, located in the bone marrow, may differentiate either into a myeloid or a lymphoid precursor, setting up the divergence between the myeloid (blue) and plasmacytoid (green) lineage. The myeloid precursor is then able to migrate into the blood stream and to differentiate into a monocyte. Monocytes migration to specific tissues and their differentiation occur upon a stimulation of a different cytokines, interleukins and/or other factors cocktail. Depending to the location, the monocytes become either interstitial dendritic cells, macrophages or microglial cells. Lymphoid precursor runs parallel with the myeloid one, but can directly differentiate into another type of dendritic cell, the plasmacytoid dendritic cell.

Cells of myeloid lineage including monocytes, macrophages and dendritic cells (figure 1) play an important role in the initial infection and therefore contribute to its pathogenesis throughout the course of infection. This is mainly because these cells are critical immune cells responsible for a wide range of both innate and adaptative immune functions.

Infected monocytes have been recovered from the blood of HIV-1 infected patients, even from those on HAART and with a viral load below detectable limits [19,31]. Early studies have shown that monocytes harbor latent HIV-1 proviral DNA [32]. Interestingly, a minor monocyte subset, the CD16+ is more permissive to the infection than the more abundant CD14++CD16- monocyte subsets [33]. Although HIV-1 proviral DNA is only in less than 1% of circulating monocytes (between 0.01 to 1%), these cells are important viral reservoirs and are responsible for the dissemination of HIV-1 into sanctuaries such as the brain [19,23,31,34,35]. Infected circulating monocytes are also recruited to the gastrointestinal tract. They later differentiate into macrophages and form the HIV-1 reservoir of the intestine [36,37]. Some authors suggest that these cells are not true latent cells, since monocytes remain in circulation for only up to 3 days and replication-competent viruses may be recovered from the blood of patients. They rather suggest that a recent ongoing infection of these cells or their precursors takes place [38]. In favor of this suggestion is the viral evolution within this compartment [19].

Dendritic cells are also involved in the dissemination of HIV-1 following primary infection [39]. After capturing viruses at the site of infection, mature dendritic cells migrate into lymph nodes where they participate in the transmission of HIV-1 to CD4+ T cells [40]. Mature myeloid dendritic cells located in lymph nodes can sustain a very low level virus replication and therefore have a potential role in HIV-1 latency and/or ongoing replication. The mechanism of this viral persistence is not yet known [41-43].

Macrophages harboring the CD4 receptor and CCR5 coreceptor are now recognized as early cellular targets for HIV-1 [44]. These cells are able to produce and harbor the virus for a longer period. This is partly due to the higher resistance of these cells to cytopathic effects. It is less clear whether macrophages have a role in HIV-1 latency [22,45] or not. In patients on HAART very few lymph node macrophages are infected (about 0,005%). However, the finding of *in vivo *reactivation of these infected macrophages in response to opportunistic infections is in favor of macrophages as HIV-1 reservoirs [46,47]. Finally, resident macrophages of the central nervous system (CNS) deserve attention since they are involved in the pathogenesis of HIV-1-associated dementia [48,49]. Four types of macrophages were described in the CNS, the meningeal macrophages, the macrophages of the choroid-plexus, the perivascular macrophages and the microglial cells [48]. Among these four types, the perivascular macrophages and the microglial cells are the main targets for HIV-1 in the CNS [49]. These cells have a low turnover, 2-3 months for the perivascular macrophages and several years for the microglial cells. These features make these cells potential reservoirs for HIV-1 [30,50].

Haematopoïtic cells (HPC) have also been proposed to serve as a viral reservoir, since a subpopulation of CD34+ HPCs express CD4 and CCR5 and/or CXCR4 and these cells are susceptible to HIV-1 infection [51-54]. Furthermore, HIV-1-infected CD34+ HPCs have been detected in some patients [55,56]. Interestingly, the CD34+ CD4+ HPC subset has an impaired development and growth when HIV-1 is present. This HPC will then generate a sub population of monocytes permissive to HIV-1 infection with a low level of CD14 receptor and an increase of CD16 receptor (CD14+ CD16++). This population of monocyte may differentiate in dendritic cells in tissues such as lymph nodes [57-59]. It is not yet well understood whether the abnormalities leading to the generation of this permissive cell population are due to a direct or an indirect interaction with HIV-1. A further investigation is needed, since these HPCs generate an infected cell lineage that may spread HIV-1 to sanctuaries.

## Mechanisms of HIV-1 latency in the monocyte-macrophage lineage

Following fusion-mediated entry into the host cell, the virus is uncoated, the virus genome is reverse transcribed and the pre-integration complex enters the nucleus where the proviral DNA is integrated into the host cell genome. In productive cells, the transcription of the provirus DNA is regulated by the interplay of a combination of viral and cellular transcription factors [60-63]. However, cells that lack or have a low level of HIV-1 expression are also present and contribute to viral persistence. It is still controversial whether or not true latency occurs in infected cells from the monocyte-macrophage lineage. For this reason, but also to avoid confusion, the word latency will be used in the following sections, not *stricto sensu *as previously defined, but in a larger sense which includes true latency and low ongoing replication. Contrary to the CD4+ T cells, in which the mechanisms of the establishment and the maintenance of true latency have been well described [64], our knowledge of the molecular mechanisms underlying latency in the monocyte-macrophage lineage is poor. Like in CD4+ T cells, two types of latency occur in cells from the monocyte-macrophage lineage.

### Pre-integration latency

Pre-integration latency is frequently observed in CD4+ T cells. This form of latency has a very limited contribution to viral persistence since the half lives of the cells is very short (1 day). On the contrary, this form of latency in the monocyte-macrophage lineage may contribute to the formation of reservoirs to a larger extent and may participate in viral dissemination. This form of latency is characterized by a poor reverse transcriptase activity and therefore it is unable to synthesize the provirus DNA. Various mechanisms are involved in this form of latency, such as hypermutation of the DNA induced by the restriction factor APOBEC3, a low level of dNTP pool and an impaired nuclear importation of the pre-integration complex associated to a low level of ATP pool [65-68]. Several reports pointed out that macrophages can harbor large quantities of unintegrated viral DNA in a circular form [69,70]. Moreover, these unintegrated DNA remain stable for up to two months in non dividing macrophages [69]. Interestingly, the accessory viral protein Vpr is important for viral replication in the monocyte-macrophage lineage, but not for non dividing CD4+ T-cells [71]. Indeed, deletion of Vpr decreases transcription from unintegrated HIV-1 DNA up to 10 times [72]. A recent report suggests that infected human macrophages can support persistent transcription from this unintegrated DNA [73]. These circular forms of episomal DNA may therefore account for persistence and expression in non dividing cells such as macrophages [74].

### Post-Integration latency

Post-integration latency occurs once the viral genome has been reverse transcribed and has been stably integrated into the host genome. At that moment, the level of transcription is very low with a no or a low level of virus replication. Mechanisms generating HIV latency in the CD4+ T cells are well described [75,76]. Viral genome integration into repressive heterochromatin may account for the establishment of latency in some cases [77]. Transcriptional interference may be responsible for the establishment of HIV-1 latency [78,79] when viral genome integrates into active euchromatin regions. Several mechanisms acting at a transcriptional and post transcriptional level that maintain the post-integration latency in CD4+ T cells have been described, but it is unknown whether these are also effective in cells of the monocyte-macrophage lineage. However, several mechanisms generating HIV-1 post-integration latency have been described in the monocyte-macrophage, including the lack of, or dysfunctional Tat, the lack of host transcriptional activators, presence of host transcriptional repressors, influence of chromatin environment and host antiviral processes such as the one based on microRNA (miRNA).

#### Mechanisms involving Tat transactivation

It has been proposed that restriction of the integrated HIV-1 genome transcription is due to the lack of Tat transactivation. The recruitment of the positive transcription elongation factor (pTEFb), which is composed of two proteins, cyclin T1 (CycT1) and cyclin dependant kinase 9 (Cdk9) [80-82] makes this transactivation effective. A lack of transactivation could be due to a low level of Cyclin T1 expression since its expression is limiting for p-TEFb function. Indeed, CycT1 is undetectable in undifferentiated monocytes but activated in monocytes-differentiated macrophages [83]. However, CycT1 is not the only limiting factor involved in the transcriptional inhibition of HIV-1. The phosphorylation status of CDK9 is also important as it increases during the differentiation process of monocytes into macrophages [84].

#### Mechanisms involving host transcriptional factors

The lack of host transcriptional activators or the presence of host transcriptional repressors may also explain latency in these cells. It has been reported that distal LTR binding sites upstream of the NF-KB binding site are essential for the efficient transcription in monocytes and macrophages. In addition to NF-KB and Sp1 binding, NF-IL6 and/or USF protein binding to the LTR modulatory region are essential for HIV-1 transcription [85-87]. In contrast, in microglial cells the core region and the NF-KB sites are sufficient for transcription [63]. Particularly, Sp1 protein plays an essential role by anchoring directly or indirectly several cellular transcription factors to the promoter, such as NF-IL6, CREB and COUP-TF [88].

The inhibiting form of C/EBPβ/NF-IL6 (LIP), a 16 kDa inhibitory isoform that is structuraly close to C/EBPγ, is expressed in macrophages during differentiation. LIP expression is linked to the suppression of HIV-1 replication [89]. Although C/EBPβ/NF-IL6 acts as an activator of HIV-1 transcription, LIP and/or C/EBPγ act as a dominant-negative inhibitor of NF-IL6 mediated transactivation [88]. Interestingly, this latter mechanism has been proposed to explain the establishment of transcriptional HIV latency in microglial cells of a macaque model, providing the first mechanism of HIV latency in the brain [90]. The TRAF signaling pathway can activate NF-IL6 via the P38-MAPK pathway and is involved in the reactivation of latently infected macrophages [91].

The zinc-finger protein OKT18, which is produced during HIV-1 infection of macrophages, suppresses HIV-1 transcription through the viral LTR [92,93]. This protein exerts its role through the suppression of Tat-mediated HIV-1 LTR activity [94] and through two DNA binding domains which have been recently identified in the LTR: The negative-regulatory element (NRE) and the Ets binding site [93]. It appears that this regulation is cell type specific since it has been reported that OKT18 expression is only detected in brain perivascular macrophages but not in microglial cells [95]. This absence of OKT18 expression in human microglial cells is due to the down regulation of YY1 and upregulation of FoxD3 following HIV-1 infection, which leads to a repression of the OKT18 promoter activity [96]. These results point to zinc-finger proteins as important modulators of HIV-1 transcription and make them attractive for devising new drugs to control AIDS [97,98]

HIV-1 transcription is also modulated by proteins of the Sp1 family which differ in the nature of the Sp protein bound to the LTR and of the cell type. Indeed, Sp1 and Sp3 are both expressed in microglial cells, unlike CD4+ T-cells, which express only Sp1. In microglial cells, although Sp1 acts as an activator of HIV-1 transcription, the Sp3 protein represses the HIV-1 promoter activity [99]. Some factors, like IL-6, or hydroxyurea could synergistically reactivate HIV-1 replication in latently promonocytic cells by increasing the ratio of Sp1/Sp3 [100]. Serpin B2, a serine protease inhibitor induced in activated monocytes and macrophages during inflammation is also able to increase the Sp1/Sp3 ratio by inhibiting Rb-degradation, and thus may reactivate latently infected cells [101].

#### Importance of the chromatin environment

It is now well established that viral promoter activity depends on the chromatin environment [102]. Nucleosomes are precisely positioned at the HIV-1 promoter [103,104]. Nuc-1, a nucleosome located immediately downstream the transcription initiation site, impedes LTR activity. Epigenetic modifications and disruption of Nuc-1 are a prerequisite to activation of LTR-driven transcription and viral expression [102]. Transcriptional repressors, like Myc bind the HIV-1 promoter and recruit histone deacetylases (HDAC) together with Sp1 and induce thereby proviral latency [105]. Recently it was shown that recruitment of deacetylases and methylases on the LTR was associated with epigenetic modifications (deacetylation of H3K9 followed by H3K9 trimethylation and recruitment of HP1 proteins) in CD4+ T lymphocytes [106]. Some studies suggest that the cellular signaling pathway which involves the receptor tyrosine kinase RON could trigger the establishment and maintenance of HIV-1 latency in monocytic cell lines. A correlation was found between RON expression and inhibition of HIV-1 transcription. Transcription was affected at different levels, i.e. chromatin organization, initiation and elongation [107-109]. The retinoid signaling pathway may also be involved in the inhibition of HIV-1 reactivation. The retinoid pathway inhibits both Nuc-1 remodeling and transcription [110].

**Figure 2:**
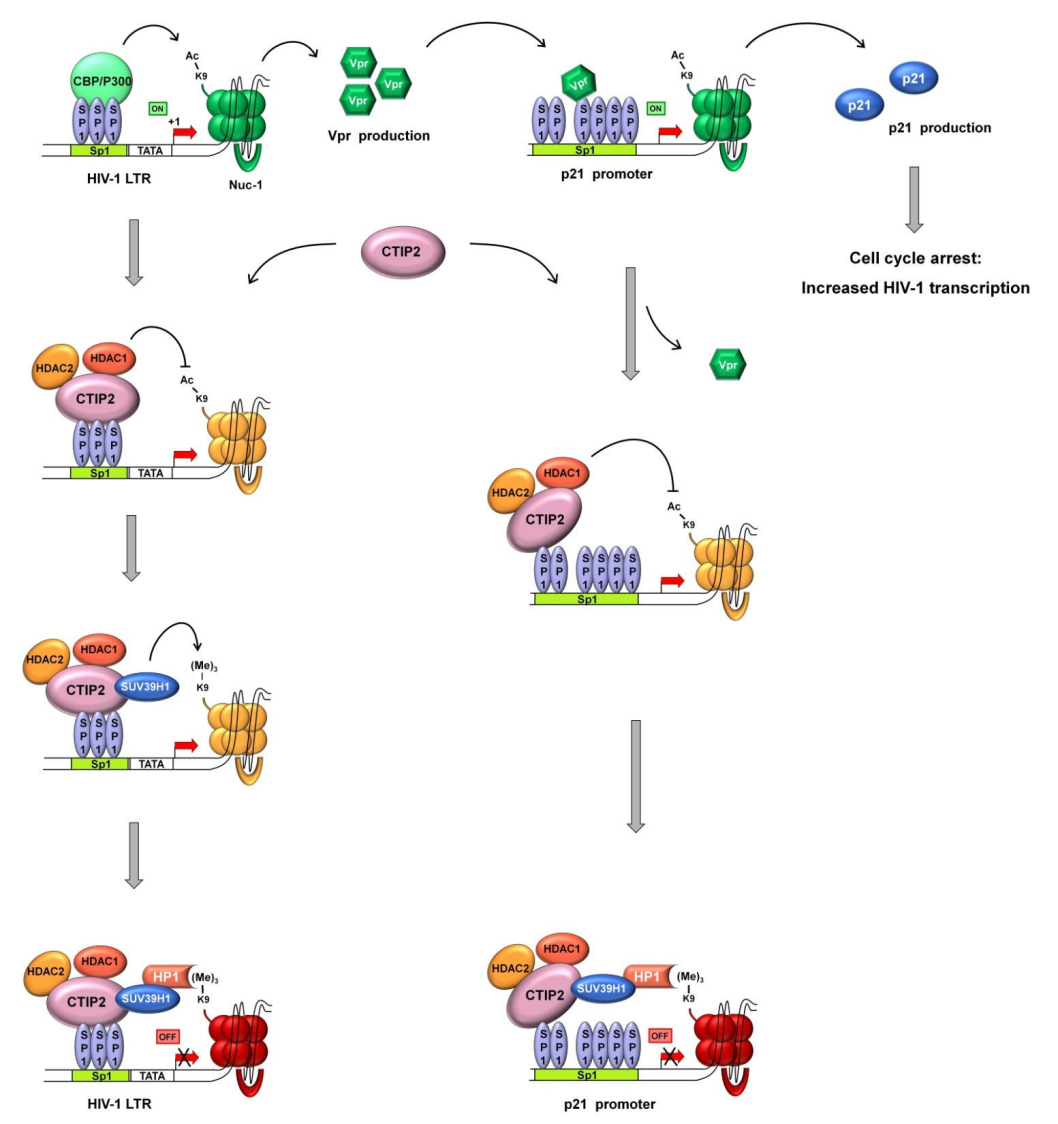
**Functions of CTIP2 in the regulation of HIV-1 gene transcription**. CTIP2 (COUP-TF Interacting Protein 2), a transcriptional repressor, has been pointed out as an actor of the latency establishment in the macrophage lineage. First, it has been shown that CTIP2 has a direct impact onto the HIV-1 LTR promoter by replacing transcriptional activator, such P300 (top left). CTIP2 interacts with Sp1, which is anchored to the LTR, switching nuc-1 from a transcriptionally active to a repressive state. Following its binding to Sp1, CTIP2 recruits sequentially histone deacetylase (HDAC) 1 and 2, which remove acetylation marks from the nuc-1 nucleosome, and then SUV39H1, which add a tri-methylation mark onto the lysine 9 of the histone protein H3. As for SUV39H1, it interacts with HP1, a protein stabilizing nuc-1 in a transcriptional closed state. Moreover, CTIP2 is also able to indirectly repress HIV-1 gene transcription. Indeed, CTIP2 can counter the action of the viral protein Vpr. One of the roles of Vpr is to induce a cell cycle arrest through activation of the p21 gene. Sp1-mediated recruitement of Vpr to the p21 gene promoter increases the production of the p21 protein, a cell cycle regulator. Consequence of such block in cell cycle is an enhancement of the viral transcription. The binding of CTIP2 to the p21 promoter forces Vpr release, HDACs and SUV39H1 recruitment, HP1 association and p21 gene silencing.

The transcription factor COUP-TF interacting protein 2 (CTIP2) has been reported [111] to play an essential role in promoting viral latency in microglial cells. This factor is a recently cloned transcriptional repressor that can associate with members of the COUP-TF family [112]. This factor is expressed in the brain and in the immune system [113]. We have previously shown that CTIP2 inhibits replication in human microglial cells [114,115]. Recently, we have shown that CTIP2 inhibits HIV-1 gene transcription through recruitment of a chromatin-modifying enzyme complex and by establishing a heterochromatic environment at the HIV-1 promoter in microglial cells [111]. Indeed, this work suggests that CTIP2 recruits histone deacetylases HDAC1 and HDAC2 to the viral promoter to promote local deacetylation of the lysine 9 from histone 3 (H3). In addition, CTIP2 has also been shown to associate to the histone methyltransferase SUV39H1, which induces trimethylation of lysine 9 from H3 therefore allowing the recruitment of heterochromatin protein 1 (HP1), heterochromatin formation and HIV-1 silencing (figure 2). Interestingly, by using a microarray analysis with a microglial cell line knocked down for CTIP2, we have shown an up regulation of the cellular cycle independent kinase inhibitor CDKN1A/p21^waf ^(unpublished data). This latter factor has been recently described as a pivotal facilitator of the HIV-1 life cycle in macrophages [116,117]. Indeed, HIV-1 infection activates p21 expression and forces a cell cycle arrest that is highly permissive for viral transcription in macrophages. We have recently reported that CTIP2 is a key transcriptional regulator of p21 gene expression [118]. CTIP2 recruited to the p21 promoter silences p21 gene transcription by inducing epigenetic modifications as described above for the HIV-1 promoter. This effect indirectly favors HIV-1 latency since activation of p21 gene stimulates viral expression in macrophages [117]. Moreover, CTIP2 counteracts HIV-1 Vpr which is required for p21 expression (see the accompanying review from Ayinde et al for more details regarding the role of Vpr in macrophage infection). We have suggested that all these factors contribute together to HIV-1 transcriptional latency in microglial cells [118]. However, p21 may have various effects along the replicative cycle of HIV-1; a very recent report from Bergamaschi et al has described p21 as an inhibitor of the HIV-1 replication [119]. Indeed, they have shown that FcγR activation can interfere with the pre-integration step of the HIV-1 cycle and is associated to the induction of p21 expression. This role of p21 as an inhibitory factor in macrophages has also been reported for other Lentiviruses such as SIVmac and HIV-2 [119]. As discussed in this latter report and in the accompanying review (Bergamaschi and Pancino), p21 might have different effects on HIV-1 infection of macrophages depending on the targeted viral life cycle step and therefore on the time since infection.

#### An original post transcriptional mechanism involved in latency: the microRNA

The host antiviral processes using microRNA (miRNA) as a defense mechanism are now considered as fundamental for regulation of animal and plant gene expression [120]. Indeed miRNA, 19-25 nucleotide long non-coding RNAs, are involved in various biological processes in eukaryotic cells [121,122]. The miRNAs interact with a complementary sequence in the 3'-UTR of target mRNAs, that leads either to mRNA degradation, or more often to translational inhibition [122]. It has been shown that miRNAs are involved as well in the regulation of virus expression [123]. These processes are manipulated or quenched by HIV-1, and this favors the establishment and maintenance of latency [124]. Recently, it was shown that miRNAs regulate the expression of the histone acetyltransferase Tat cofactor PCAF, and HIV replication [125]. Moreover, an enrichment of miRNAs in clusters has been observed only in resting CD4+ T cells and not in active CD4+ T cells [126], suggesting that these miRNA clusters inhibit HIV replication and therefore contribute to HIV latency in resting primary CD4+ T cells. A similar mechanism, based on cellular miRNA, has also been described in circulating monocytes [127]. In another recent report Sung & Rice have identified a miRNA (miR-198) that is strongly down-regulated when monocytes are induced to differentiate. Moreover, they have shown that this miRNA restricts HIV-1 replication through the repression of CycT 1 expression [128]. This result confirms previous observations that translation of CycT1 mRNA is inhibited in monocytes [129]. Identification of additional miRNAs involved in the repression of host and/or viral factors that could be involved in HIV-1 restriction are needed. Altogether, these data indicate that miRNA are crucial in promoting HIV-1 latency and suggest also that a manipulation of miRNAs could be useful in therapies aiming to purge reservoirs [130].

#### Influence of the microenvironment in establishing latency

Finally, it has been proposed that the establishment of latently infected macrophages occurs in a suppressive microenvironment made of apoptotic cells [131]. Apoptotic cells induce an inhibition of HIV-1 transcription in the infected macrophages by a signal transduction which involves ELMO. This molecule is indeed involved in the phagocytosis of apoptotic cells [131].

## Mechanisms of HIV-mediated apoptosis resistance in the monocyte-macrophage lineage

Another strategy developed by the virus in order to persist in infected cells is to render them resistant to apoptosis.

### The NF-kB pathway

Several reports have pointed out that NF-kB activity prevents cells to undergo apoptosis [132,133] The pathway involving NF-kB is activated upon HIV-1 infection in monocyte cells and in primary macrophages [134] (see also the accompanying review from Herbein et al). It has been proposed that TNFα-induced NF-kB activity might be involved in the inhibition of apoptosis and the survival of monocytes and macrophages even if Tumour Necrosis Factor alpha (TNFα) is best known as a pro-inflammatory mediator capable to induce apoptosis. Persistent HIV-1 infection of macrophages results in increased levels of the transcription factor nuclear factor kappa B (NF-κB) in the nucleus secondary to increased IκBα, IκBβ, and IκBε degradation, a mechanism postulated to regulate viral persistence [135,136]. NF-κB is involved in the resistance to TNF-induced apoptosis that might result in a decreased susceptibility to apoptosis of macrophage versus T cells in the context of chronic immune activation like in HIV-1 infection. This indicates clearly that HIV-1 can manipulate the apoptotic machinery to its advantage. Moreover, HIV-1 can induce a dual regulation of the anti-apoptotic protein Bcl-2, resulting in persistent infection of monocytic cells [137]. HIV-1 infection first results in a decrease of Bcl-2 and thioredoxin, permitting an initial boost of replication. Then, as the synthesis at the transcriptional level proceeds, replication is negatively controlled by Bcl-2 to reach a balance characterized by low virus production and higher Bcl-2 and thioredoxin levels resulting in low but sustained viral production compatible with cell survival [137,138]. Recently, the absence of apoptosis in HIV-1-infected primary human macrophages has been reported to correlate with an increase in anti-apoptotic Bcl-2 and Bcl-XL proteins and a decrease of pro-apoptotic Bax and Bad proteins [139].

### The role in apoptosis of viral proteins is often dual

The protein Nef is a regulating protein expressed early and abundantly throughout the course of HIV-1 infection. This protein has dual effects depending on the stage of infection. In the early stage, Nef contributes to the constitution of reservoirs with sustained virus production. It mimics the action of TNFα with subsequent activation of NF-kB and MAPK [140,141]. In latter stages, it is involved in the inhibition of apoptosis in infected cells by blocking TNF-mediated apoptosis [142-144]. The Nef protection to the HIV-1-induced apoptosis correlated with the hyper-phosphorylation and consequent inactivation of the pro-apoptotic Bad protein [143]. Finally, Nef is also involved in the blockade of p53-mediated apoptosis [145]. Therefore the Nef anti-apoptotic effect could be a relevant part of the mechanism of the *in vivo *establishment of the HIV-1 macrophage reservoirs. Macrophage express 10-times lower numbers of cell surface CD4 than CD4^+ ^T cells [146], and therefore might be less susceptible to HIV-1 superinfection. Since high levels of cell surface CD4 on HIV-infected cells reportedly induce a dramatic reduction in the infectivity of released virions by sequestering the viral envelope by CD4 [147], low levels of CD4 on the cell surface of macrophage might favour the release of infectious virions from the infected cell, and thereby could optimize transmission of virions to the cells present in the vicinity. Third, the viral life cycle of HIV-1 is 6-times slower in primary macrophage than in primary T cells due to a slower reverse transcription process, suggesting that the rate of virion production might be lower in macrophage than in CD4^+ ^T cells, thereby allowing macrophage to form long-lasting virus reservoirs [148,149].

Tat and gp120 have also dual effects on apoptosis depending of the cell type. In the central nervous system, HIV-1 triggers apoptosis in neurons. This is also seen when neurons are exposed to extracellular Tat or gp120 [150]. On the other hand, microglial cells, the CNS resident macrophage, do not undergo apoptosis upon HIV-1 infection or following exposure to extracellular viral proteins such as Tat or gp120 [151-153]. Tat-mediated resistance to apoptosis in microglial cells is due to the activation of the PI-3-K/AKT cell survival signaling pathway. The protein Tat also decreases the activity of p53. The protein Tat has also been shown to mediate apoptosis resistance by up regulating Bcl-2. This anti-apoptotic factor inhibit TNFα related apoptosis-induced ligand (TRAIL mediated apoptosis) [154]. This combined action of Tat will therefore favor long term cellular survival observed in microglial cells throughout the course of HIV-1 infection [152].

Over-expression of CTIP2, described as an anti-apoptotic factor [155], in microglial cells leads to a repression of p21 expression [118]. This is partly due to the inhibition of the p53 activity on p21 transcription and also to the fact that CTIP2 counteracts Vpr. This latter protein has been shown to trigger apoptotic events in infected lymphocytes and in neurons [156-158]. Taken together, the data suggest that CTIP2 might be involved in the apoptosis resistance of microglial cells, besides its role in the establishment and maintenance of HIV-1 latency. Some investigators have shown that p21 transcription is slightly increased in monocytes recovered from chronically-infected patients and is associated with an anti-apoptosis signature [159]. The apparent discrepancy in the role of p21 in apoptosis in monocytes versus microglial cells needs to be clarified. It might arise from the dependence of the activities of p21 on the cell type, subcellular location, expression level and phosphorylation status. Moreover, p21 expression is regulated by both p53-dependent and p53-independent mechanisms. An increase in p21 expression mediated by Fcγ R activation in macrophages was not due to an induction of p53 since its silencing did not block p21 induction by Immune Complexes [119]. It should be noted that it is not clear whether p21 is an oncogene [160] (which could be involved in the inhibition of apoptosis) or an anti-oncogene [161] (which could be involved in the induction of apoptosis).

The protein gp120 produced by monocyte-macrophages inhibits TRAIL-mediated cell death by inducing the expression of macrophage-stimulating factor (M-CSF). This envelope protein also up-regulates expression of several anti-apoptotic genes such as Bfl-1 and Mcl-1 [162]. A stable signature of anti-apoptosis, comprising 38 genes including p53, MAPK and TNF signaling networks has also been identified from circulating monocytes of HIV-1 infected patients [159]. CCR5 co-receptor bound by HIV-1 can lead to apoptosis resistance in monocyte cultures. A recent report has also stressed the central role of CCR5 during HIV-1 infection [163]. This paper described a case of a HIV-1 positive patient, who received bone marrow transplantation for leukemia. In the follow-up study there was no evidence of the virus in the bloodstream even after 20 months. Myeloablation and T cells ablation were suggested to favor the elimination of the long-lived reservoirs. Indeed, transplantation was done with cells from a homozygous donor for mutation in the HIV-1 co-receptor CCR5. This mutation is well known to be associated with resistance to HIV-1 infection. Therefore development of new molecules to inhibit CCR5 coreceptor function will be a great challenge in the next years. It will be also interesting to investigate whether the interaction between CXCR4 co-receptor and HIV1 could also trigger apoptosis resistance. Apart from the critical role CCR5 plays in maintaining HIV-1 infection, this study also raises the possibility that the main target to cure the patients from AIDS are the peripherical circulating cells including the monocytes (and by the way the infected HPCs). Indeed, whole-body irradiation leading to complete remission of acute myeloid leukemia will mainly target radiosensible cells such as HPCs and peripherical circulating cells. The fact that no virus has been detected at month 20 of follow-up, might suggest that reservoirs in sanctuaries could not sustain viral replication alone. However, the importance of these reservoirs in the physiological context of infected WT-CCR5 patients should not be neglected.

### Discovery of a new anti-apoptotic mechanism based on miRNA

Finally, a new mechanism has been proposed that protect cells from apoptosis and therefore extend the lifespan of infected cells. This mechanism is based on the suppression of the cell's RNAi activity by synthesis of a TAR miRNA, a small hairpin RNA. This RNA is capable to sequester the miRNA processing machinery of the cell and therefore impedes the functioning of cellular antiviral miRNAs [164]. Moreover, this TAR miRNA has also been shown to be involved in the down regulation of the expression of several proteins related to apoptosis [165].

Our knowledge of the mechanisms involved in apoptosis resistance is far from complete. The diversity of strategies used by HIV-1 to manipulate the apoptotic pathway emphasizes the capacity this virus possesses to survive in its host. We note that mechanisms involved in apoptosis resistance, at least the mechanisms involving the TNF-signaling pathway, are also involved in virus production. It seems that the nature of this reservoir is rather different from the latent reservoir. The therapeutical implications are therefore important since stimuli such as phorbol esters will not be suitable to purge the reservoir. Indeed, this treatment will reactivate the expression of HIV-1 in latent reservoir but may increase the resistance to apoptosis in viral reservoir that exhibit a sustained production of virions. The survival of viral reservoirs is of great importance since it is also an obstacle to HIV-1 eradication. The mechanisms underlying this apoptosis resistance are essential for devising new and original therapeutic strategies to purge the reservoirs, but are far from being completely known.

## Implications for therapy

The introduction of HAART in 1996 has greatly improved survival, but it has been unable to eradicate the virus from latently infected reservoirs. A principle cause may be that besides the best characterized cellular reservoir of memory CD4+ T-cells there are other reservoirs, such as the monocyte-macrophage reservoir. Moreover, these cells are often found in tissue sanctuary sites, like the brain, that are protected from drug penetration [166-168]. Furthermore, several reverse transcriptase inhibitors are ineffective in chronically infected macrophages [34] and protease inhibitors have significantly lower activities in these cells compared to lymphocytes [169]. The emergence of multidrug resistant viruses has been reported in an increasing number of patients receiving HAART [110,170,171]. Finally, the nature of the reservoirs (latent reservoir with no or low virus replication versus productive reservoir which are resistant to apoptosis) has to be taken into account. These considerations (existence of several reservoirs, tissue-sanctuary sites and multidrug resistance) encourage the search for new and original anti HIV-1 treatment strategies. New methods should be developed which target each of these reservoirs. We believe that eradication of the virus could be achieved by specifically purging targeted reservoirs and concomitantly eliminating the virus by a reinforced HAART. Another way to control HIV-1 replication is to re-inforce the latency status by using transcriptional inhibitors.

### Use of transcriptional inhibitors to control HIV-1 progression

At present the therapy of HIV-1-infected patients is based on a combination of HIV gp41, reverse transcriptase and protease inhibitors. We believe that new drugs should target other steps of the HIV-1 cycle. For example, they could be directed against proteins involved in the transcription of the inserted virus genome. Tat has a critical role in transcription, and constitutes a major target in therapeutic intervention in the HIV replicative cycle [172-174]. Moreover, drugs could be designed to target cellular cofactors involved in the activation of transcription. This strategy should be able to by-pass drug-resistance which arises with viral proteins. Therefore, ways to synthetize drugs which interfere with HIV-1 replication in monocyte-macrophage should be devised [175]. Several transcriptional inhibitors already characterized such as C-terminally truncated STAT5, Staf 50, Prothymosin α and thioredoxin reductase [176-179] could be used for controlling viral expression in the human macrophages. Inhibition of the NFAT5-LTR interaction by using small interfering RNA is also promising since it suppresses HIV-1 replication in primary macrophages and therefore progression of AIDS [180]. The discovery that only macrophages are able to repress HIV-1 transcription and replication in response to Il10 need further investigation since we could specifically control HIV-1 expression in these cells [129,181]. The demonstration that treatment of HIV-1 infected lymphocytes with the O-GlcNAcylation-enhancing agent glucosamine repressed viral transcription opens the way to metabolic treatment [182]. This treatment might work in the monocyte-macrophage lineage since this chemical compound affects Sp1 and therefore inhibits the activity of the LTR promoter. According to several reports, OKT18, a zinc-finger protein, can reduce HIV-1 replication in human macrophages by the suppression of Tat-induced HIV-1 LTR activity [93,183]. New approaches based on engineered transcription factors are now emerging with zinc finger protein as an attractive candidate for antiretroviral therapy since their binding to HIV-1 LTR in a sequence specific manner is associated with the repression of LTR activity [97,98]. Interestingly, zinc-finger protein can influence the chromatin compaction and nuclear organization through regulation of proteins involved in epigenetic regulation [98].

Finally, new drugs must be designed with properties that allow them to penetrate tissue-sanctuary sites such as the brain [166].

### Strategies based on virus reactivation from latent reservoirs

**Figure 3:**
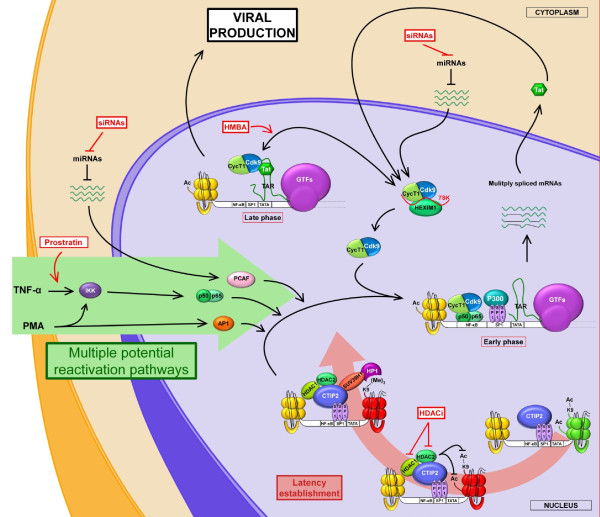
**Pharmaceutical approaches of the potential reactivation pathways on latently integrated HIV-1 genome**. Multiple ways of reactivation are possible to occur to re-initiate the HIV-1 transcription. Extern signals, such as TNF-α, can trigger the activation of transcriptional activator, like the heterodimer p50/p65. In the mean time, host protein balance may change, leading to higher availability of transcriptional activators. For instance, miRNAs regulates the rate of PCAF, a coactivator produced by the host cell (green arrow - Multiple potential reactivation pathways). There are some critical steps in this process that may be targeted to reactivate or hinder the latency establishment (Red boxes). HDAC inhibitors (HDACi) may prevent the formation of heterochromatin; Prostratin induces the IKK activation, which provokes the activation of transcriptions factors; HMBA increases the pTEFb release from the inactive stock; it is possible to reverse the miRNAs negative impact on the mRNAs of transcriptional activators and/or CycT1 through specific siRNAs.

Recently, a new and original strategy has been proposed to eradicate the virus from infected patients. The main idea is to facilitate the reactivation of viruses from latent reservoirs, which are then destroyed by HAART (figure 3). Many factors have been involved in reactivation including physiological stimuli, chemical compounds like phorbol esters, histone deacetylase inhibitors, p-TEFb activators, and some activating antibodies (antiCD3). Many eradication protocols passed preclinical studies [2] but to date all failed in clinical trials. Some protocols failed due to the potential toxicity of treatments based on non specific cell activation such as IL2 [184]. The recent discovery that an alternatively spliced form of the cellular transcription factor Ets-1 can activate latent HIV-1 in an NF-KB independent manner has highlighted the therapeutic potential of cellular factors for the reactivation of latent HIV-1 [185]. Future eradication protocols should use a combination of drugs targeting all the viral silencing mechanisms identified to reactivate HIV-1 from latently-infected cells. A protocol with a similar approach gave promising results. In this study, the association of a HDAC inhibitor or a DNA methylation inhibitor with prostratin (phorbol ester that stimulates protein kinase C activity) proved to have a synergistic effect on the activation of HIV-1 expression [186,187].

### Strategies based on increasing apoptosis susceptibility

Increasing the susceptibility of the infected cells to apoptosis is also of great interest. Essentially, the HIV-1 proteins Tat, Nef and the envelope protein gp120 should be targeted, as these proteins have a crucial function in different steps of the virus cycle and also in the acquired resistance to apoptosis. A better comprehension of mechanisms involved in resistance to apoptosis has also allowed to devise drugs against host factors which render cells susceptible to die. For example, molecules which interfere with the chemokine receptor CCR5 are promising since these molecules are both involved in virus entry and in apoptosis resistance. Several CCR5 antagonists are already used in clinical trials [188,189]. A chemotherapeutic drug, Imatibib, restored apoptotic sensitivity of HIV-1 macrophages through the inhibition of the activity of the pro-survival cytokine macrophage colony-stimulating factor (M-CSF) [162]. Finally, several AKT inhibitors including Miltefosine are also promising molecules for targeting long-lived viral reservoirs [190].

A dramatic reduction of monocyte-macrophage reservoirs might be achieved by strategic interventions targeting both the resistance of infected cells to apoptosis and the reactivation of latently-infected cells associated with a reinforced antiretroviral therapy.

## Conclusion

Cells from monocyte-macrophage lineage are an ideal reservoir for HIV-1. This is due to several features of these cells including long lifespan, absence of direct cytopathic effect, apoptosis resistance of infected cells, existence of latently infected cells (with low or no virus expression) and their localization in sanctuaries. The purging of this reservoir is therefore crucial since it constitutes one of the major obstacles to HIV-1 eradication from infected patients. A drastic reduction of this reservoir might be achieved by the development of new and original therapies which target specifically all the reservoirs, including the monocyte-macrophages. These treatments might not lead to a complete eradication of HIV-1 but there is hope that a drastic reduction could be achieved with levels of expression comparable to those in elite non progressors patients who remain asymptomatic without an antiretroviral therapy.

## Competing interests

The authors declare that they have no competing interests.

## Authors' contributions

CS conceived and wrote the manuscript. VLD, GH and OR contributed to the writing of the manuscript. VLD created figures 1, 2 and 3 and their legends. All authors have read and approved the final manuscript.
